# Supplementary material for: Touch inhibits touch: sanshool-induced paradoxical tingling reveals perceptual interaction between somatosensory submodalities
Source: Proc Biol Sci. 2021 Jan 27;288(1943):20202914. doi: 10.1098/rspb.2020.2914 (PMC7893281; doi:10.1098/rspb.2020.2914)
Supplement: Supplementary Material [file rspb20202914supp1.docx]

**Supplementary Material for:**

**Touch inhibits touch: sanshool-induced paradoxical tingling reveals perceptual interference between somatosensory submodalities**

Antonio Cataldo, Nobuhiro Hagura, Yousef Hyder & Patrick Haggard

**Supplementary Methods**

***Experiment 1***

We tested whether the intensity of tingling sensation induced by sanshool is modulated by the application of sustained touch.

The target sample size for this experiment (n = 10) was defined a priori, based on comparable previous experiments [1,2].

***Experiment 2***

In Experiment 1, the location where the participants rated the tingling sensation was fixed to one location. In Experiment 2, we confirmed that the result of Experiment 1 is not due to sustained attention to a fixed location of the lips, by varying the location of touch, as well as the location the participant rated the tingling intensity.

The target sample size for this experiment (n = 10) was defined a priori, based on comparable previous experiments [1,2].

***Experiment 3***

In Experiment 3, we tested whether the inhibition of tingling sensation is modulated by the strength of the sustained pressure.

The target sample size for this experiment (n = 8) was defined a priori based on comparable previous experiments [1,2]. One participant could not follow the instruction of keeping the lips apart (see below), and was therefore replaced.

***Experiment 4***

In Experiment 4, we quantified the inhibition of tingling by the sustained pressure by means of amplitude of RA range vibration, and we investigated the temporal dynamics of SA1-RA interaction.

The target sample size was calculated a priori based on the power analysis using an estimate of effect size for attenuation of tingling taken from Experiment 3 (alpha = 0.05; desired power = 0.95; η_p_^2^ = 0.6; estimated sample size n = 12). Eighteen participants originally volunteered for this study. However, two participants could not keep their lips apart, and another two could not perceive strong tingling sensation by sanshool. These participants withdrew from the study, and therefore are not included in the final sample size (n = 14).

In the initial inspection of the data, we found that the distribution of the amplitude data was significantly deviated from the normal distribution (see Supplementary Table S7). Therefore, the statistical analysis was conducted by log-transforming the data. However, to maintain the data in interpretable scale, we plot the means and the standard errors in the original units (μm).

***Control Experiment 5***

Control Experiment 5 investigated the contribution of C-nociceptive fibers to the tingling perception induced by Sanshool.

***Participants***

Eight participants took part in Experiment 5 (age range: 18-38 years; 7 females). Given the design implemented (see below), participants were only included if they satisfied all of the following conditions: 1) clear sensation of tingle evoked by sanshool; 2) clear nociceptive sensation evoked by noxious electrocutaneous stimulation; 3) effective lidocaine block of nociceptive afference, as measured by reduced pain sensation/increased pain threshold. Of the 14 participants originally recruited, one was excluded during the pre-test evaluation of sanshool-induced tingling, because administration of sanshool did not elicit any tingling on the lips after 10 minutes from application; another was excluded during the pre-test evaluation of pain thresholds, because electrocutaneous stimulation within the safe intensity range did not elicit any painful sensation. Four further participants were excluded in the post-test evaluation of pain, because they did not show any numerical increase in pain threshold after lidocaine administration.

Given the Bayesian analyses approach used in this study, and the desirability of avoiding unnecessary painful stimulation, data collection was interrupted after reaching the minimal significant result supporting either hypothesis.

All participants were naive regarding the experimental purpose and gave informed written consent. Methods and procedures were approved by University College London Research Ethics Committee. Experiment 6 was approved by the Research Ethics Committee of the School of Advanced Study, University of London.

***Rationale***

Experiment 5 investigated whether sanshool-induced tingling might reflect the activity of the nociceptive channels induced by the activation of nociceptive small-diameter C-fibres. Animal studies show that sanshool activates both large-diameter myelinated (Aβ) neurons, as well as small-diameter unmyelinated C-fibres [3–5]. Although nociceptive Aβ neurons have also been recently described in humans [6], it is not clear whether these fibres are also activated by sanshool. We reasoned that attenuation of sanshool tingling by steady pressure could only be considered a touch-touch interaction if the tingling sensation could be attributed to a perceptual channel for touch, rather than a nociceptive channel. Therefore, to rule out the contribution of nociceptive C-fibres to sanshool tingling, we measured both pain thresholds and perceived intensity of tingling before and after blocking the activity of small fibres through lidocaine [7,8]. If the tingling is unrelated to the C-fibre activation, the sensitivity to pain would be affected by lidocaine, but the intensity of the tingling sensation would not.

***Experimental design and Procedure***

Sanshool (20% solution) was applied on the lower lip. Pain thresholds for electro-tactile stimuli delivered on the lips were obtained using 4 mm diameter concentric bipolar electrode connected to a constant current stimulator (Digitimer, Ltd., DS7, Welwyn Garden City, United Kingdom) (Supplementary Figure S5A). This type of electrode is known to preferentially activate nociceptive fibres at low intensities [9]. Small diameter C-fibres were blocked through a 0.9%w/w lidocaine hydrochloride solution (Boots UK Ltd, ANBESOL liquid, Nottingham, United Kingdom), a non-prescription topical anaesthetic widely used for relief of orofacial pain. Lidocaine is thought to affect predominantly Nav1.7 channels [8] and to preferentially block nociceptive fibres [7] . While lidocaine also blocks large fibres [7,10], its anaesthetic effects on different submodalities display a distinct temporal gradient [10]. In particular, pinprick and thermal sensations that characterise nociceptive and thermoceptive activities are impaired within 10 minutes of application. Conversely, tactile, proprioceptive, and motor sensations associated with larger fibres activity are affected only after longer intervals after administration (~15 minutes) [10]. Thus, to ensure an effective block of smaller fibres only, tests were performed within 10 minutes of lidocaine application.

In a 2 (time: pre-block, post-block) x 2 (sensation judged: tingling, pain) within-subject design, pain thresholds and tingling intensity ratings were measured before and after administration of lidocaine. Participants performed two sessions. In session A, participants rated the intensity of sanshool tingling on the lower lip (from 0: “no tingling at all”, to 10: “the strongest tingling sensation imaginable”). Session B took place at least one hour after session A, when participants confirmed that the tingling sensation had completely disappeared [11]. First, participants’ pain thresholds were estimated using a staircase procedure [12] (see Supplementary Material for details), then lidocaine was applied on a 2 x 1 cm area in the centre of the lower lip. Pain thresholds were estimated again after three minutes. Immediately after the second pain threshold estimate, the same area of the lip was painted with sanshool, and participants rated the intensity of tingling, using the same scale used in session A. The table of individual data for Control Experiment 5 can be found in the Supplementary Table S5.

***Control Experiment 6***

Control Experiment 6 investigate the contribution of C-tactile fibers to the tingling perception induced by Sanshool.

***Participants***

The experiment took place in the context of a science exhibition at the Tate Modern (London). A total of 64 attendees took part in the event. However, only the data from participants respecting all the following inclusion criteria were used for further analysis: 1) being between 18 and 65 years old; 2) not wearing any lipstick, lip gloss, or lip balm; 3) feeling a clear tingling sensation evoked by sanshool (i.e. baseline rating ≥ 3); 4) completing all the trials of the experiment. This left a final sample size of 51 participants (31 females). All participants were naive regarding the experimental purpose and gave informed written consent. Methods and procedures were approved by the Research Ethics Committee of the School of Advanced Study, University of London.

***Rationale***

Experiment 6 investigated whether sanshool tingle might reflect activation of C-tactile afferents. Small-diameter C-fibres responsive to tactile, but not to nociceptive stimuli have been characterised in detail by many animal [13,14] and human studies [15,16]. Although C-tactile fibres are commonly found in hairy, but not glabrous skin [16], there is electrophysiological evidence of the existence of C low-threshold mechanoreceptors in the glabrous skin of rat hind paw [17], and psychophysical evidence, using nerve blocks, of a C low-threshold input from the glabrous skin of human hand [18,19]. While we are not aware of reports of C-tactile fibres innervating the skin of the lips, this may simply reflect previous sampling, and the possibility cannot be excluded. C-tactile fibres generally respond preferentially to tactile stimuli moving at intermediate velocities [15,16]. Tactile motion tuning cannot readily be assessed with a chemical stimulus like sanshool. Importantly, however, C-tactile fibres also show preference for neutral, skin temperature (32 °C) stimuli, rather than warm (40 °C) or cold (18 °C) stimuli [15]. Thus, if the sanshool tingle is mediated by a C-tactile channel, the perceived intensity of sanshool tingling should be maximal at neutral temperatures and reduced during cold or warm thermal stimulation, producing an inverted U-shape.

***Experimental design and Procedure***

The perceived intensity of sanshool-induced tingling on the lower lip (20% solution) was assessed during three different thermo-tactile conditions: cold (21 °C), neutral (33 °C), and warm touch (41 °C) (Supplementary Figure S5B). As a baseline condition, tingling intensity without any stimulation was also measured using the same scale used in Experiment 5. Thermo-tactile stimuli were delivered using a 13 mm diameter Peltier thermode (Physitemp Instruments Inc, NTE-2A, New Jersey, USA). Each trial started with a 10 s countdown to allow the thermode to reach the intended temperature. Then participants applied their lower lip against the thermode probe. Participants were asked to move their head to approach the probe of the Peltier device in each trial until their lower lip contacted it, and then maintain a posture that applied gentle touch for 4 s. The experimenter monitored that attendees kept their lip in stable contact with the stimulator for the entire duration of the stimulation. Four seconds after the initial contact, participants were prompted to rate the intensity of the tingle. After the rating, participants withdrew their lip from the thermode. Each thermo-tactile condition was repeated four times (12 trials in total). The order of thermal conditions was randomised across participants. The table of means for Control Experiment 6 can be found in the Supplementary Table S6.

**Supplementary Results**

***Experiment 1.*** ***Spatial gradient of the tingle inhibition by probe touch***

To investigate the spatial gradient of the tingle inhibition by probe touch, we directly compared the tingle ratings across different touch locations on the upper and lower lip. 2 (lip; target position same or different to the probe touch) x 3 (position; three horizontal locations on the lip) ANOVA revealed significant main effect both for the factor of the lip (F(1,9) = 20.33, *p* = 0.001, η_p_^2^ = 0.693) and the position of the probe touch (F(2,18) = 8.43, *p* = 0.003, η_p_^2^ = 0.484). An interaction effect was also significant (F(2,18) = 5.28, *p* = 0.016, η_p_^2^ = 0.370). To investigate the nature of the interaction effect, ratings were also analysed within each lip. One-way ANOVA on ratings of lower lip touch (positions 5, 6 and 7) revealed significant main effect between the three different probe positions (F(2,18) = 10.89, *p* < 0.001, η_p_^2^ = 0.547). Planned comparisons showed that tingle intensity was reduced more strongly by pressure at position 6 (target position) than at position 5 (t(9) = 3.05, *p* = 0.014, dz = 0.96) and position 7 (t(9) = 4.32, *p* = 0.002, dz = 1.37) . For the upper lip (positions 2, 3 and 4), although the average rating was numerically lower for the middle position (position 3; 74.2% of baseline intensity) it was not significantly different from the adjacent two locations (position 2; 81.2%, position 4; 79.2%) (F(2,18) = 10.868, *p* = 0.55, η_p_^2^ = 0.065).

***Control Experiment 5. Sanshool-evoked tingling is not mediated by small-diameter unmyelinated C-nociceptive fibres***

Bayesian statistics [20,21] were used as the experiment was designed to test the null hypothesis (i.e., lidocaine does not reduce sanshool tingle). First, we ran a one-tailed Bayesian t-test to confirm that lidocaine effectively blocked small C-nociceptive fibres. As expected, participants’ pain thresholds were significantly higher after lidocaine gel administration to the lips (mean 1.08 mA ± SD 0.27), compared with pre-administration (mean 0.84 mA ± SD 0.27) (BF_10_ = 18.26; error % < 0.001) (Supplementary Figure S6A). We then tested our null hypothesis that lidocaine administration would not affect sanshool-induced tingling. As the alternative hypothesis (i.e. lidocaine reduces tingling) was unidirectional, a one-tailed test was used. The Bayesian analysis showed that the data were more likely under the null than under the alternative hypothesis (BF_01_ = 3.239; error % = 0.009). Tingling ratings were statistically identical before (mean 5.78 ± SD 1.7 arbitrary units) and after (mean 5.81 ± SD 1.5 arbitrary units) lidocaine administration (Supplementary Figure S6B).

Thus, despite effective block of nociceptive afference by lidocaine, sanshool-evoked tingle remained unaltered, suggesting the small fibre C-nociceptors are not the main contributor on tingling sensation. Therefore, tactile gating of tingle presumably involves a different mechanism to the familiar “gate control” of pain by touch.

***Control Experiment 6. Affective touch channel activation does not mediate sanshool-evoked tingling***

First, we confirmed that sanshool tingling is suppressed by the sustained touch, as shown in Experiments 1-4. As expected, sustained touch at neutral temperature significantly decreased (53.8%) the sanshool tingling intensity (mean rating 2.81 ± SD 1.7) compared with no mechanical touch (mean rating 6.08 ± SD 1.9) (t(50) = 9.01, *p* < 0.0001, dz = 1.26) (Supplementary Figure S6C). Next, we compared ratings of tingling intensity under different temperature conditions. Cold touch produced the lowest ratings (1.85 ± SD 1.7), and warm touch the highest (4.67 ± SD 2.6) (Supplementary Figure S6C). Therefore, the suppression effect decreased as the temperature of the stimulus increases. We accordingly found a significant main effect of temperature conditions in a one-way repeated measures ANOVA on participants’ intensity ratings of tingling during the three thermo-tactile conditions (F(1.5, 77.2) = 43.34; *p* < 0.0001; η_p_^2^ = 0.464). All pairwise comparisons were significant (*p* ≤ 0.002 in each case; Bonferroni-corrected). This suppression pattern clearly differs from the inverted U-shape that would be expected from C-tactile fibre thermal sensitivity. On the other hand, the linear relation to temperature is consistent with the known thermal modulation of SA fibres, which respond more at lower temperatures (see Discussion).

**Supplementary Figures**


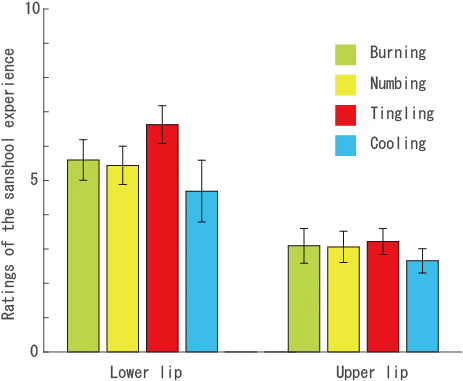


**Supplementary Figure S1. Manipulation check to confirm effect of sanshool solution strength on sensations in Experiment 3.** 80% solution of sanshool was applied to the lower lip, whereas 20% solution of sanshool was applied to the upper lip, aiming to induce stronger intensity of tingling experience on the lower lip compared to the upper lip. Participants reported the sanshool induced tingling experience using numerical ratings (0 to 10, 0: no sensation, 10: strongest imaginable sensation). They were also asked to report other sensations which have been associated with sanshool; burning, numbing and cooling (Hagura et al., 2013). For all the descriptors, the lower lip had higher ratings. Tingling sensation, which is our main interest, was significantly stronger for the lower lip than the upper lip (t(7) = 6.94, p < 0.001, dz = 2.45). This shows that our manipulation to induce different levels of tingling intensity using different solution strengths on upper and lower lip was successful. Error bars indicate standard error of the mean across participants (n = 8).

**
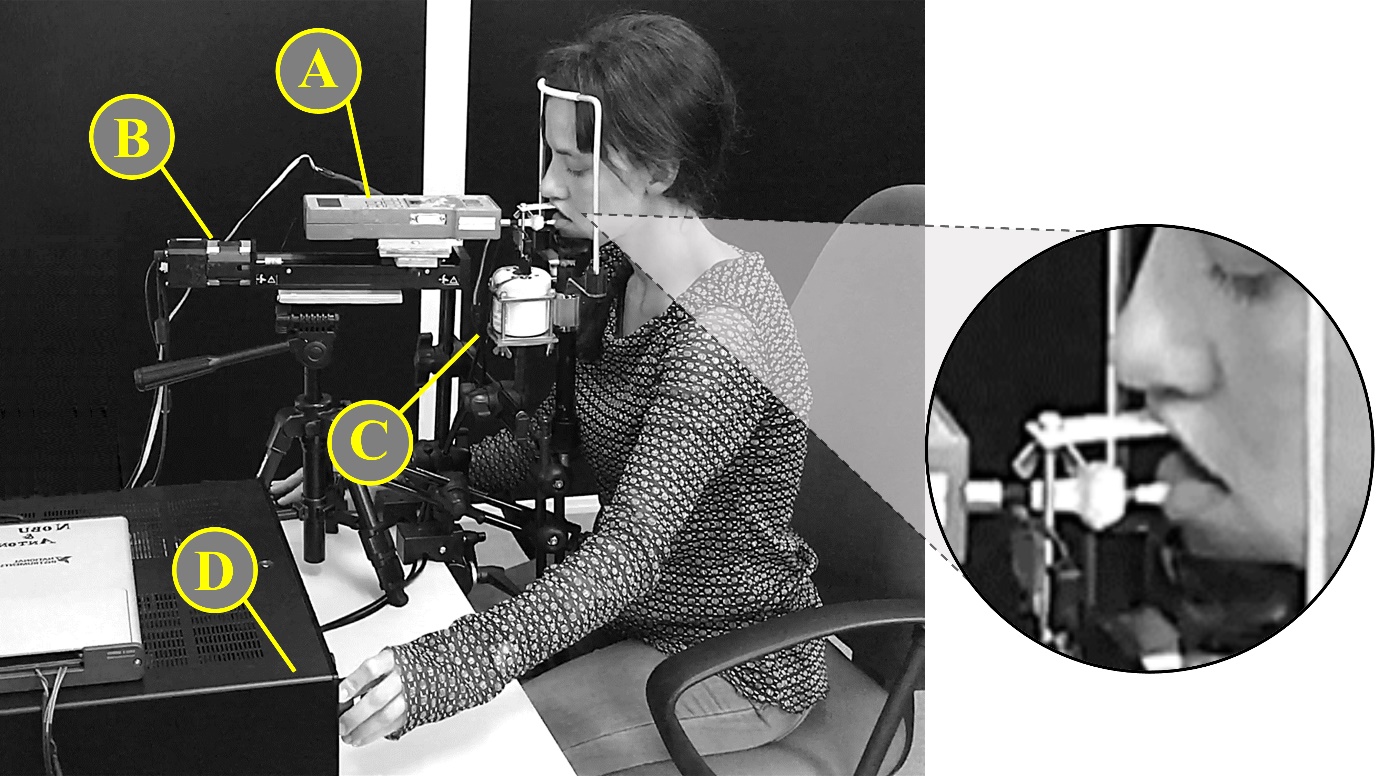
Supplementary Figure S2. Experimental setup in Experiment 3 and 4.** In Experiments 3 and 4, sustained touch was applied to the lower lip by a mechanical probe, which exerted a target level of contact force via a motor (**B**) controlled in a closed-loop arrangement using a strain-gauge force sensor (**A**). In Experiment 4, 50 Hz mechanical vibration was applied to the upper lip by a vibrator (**C**). Participants could adjust the vibration amplitude using the gain knob of an amplifier **(D**). See Supplementary Video S1 for a video of the setup and an example trial of Experiment 4.


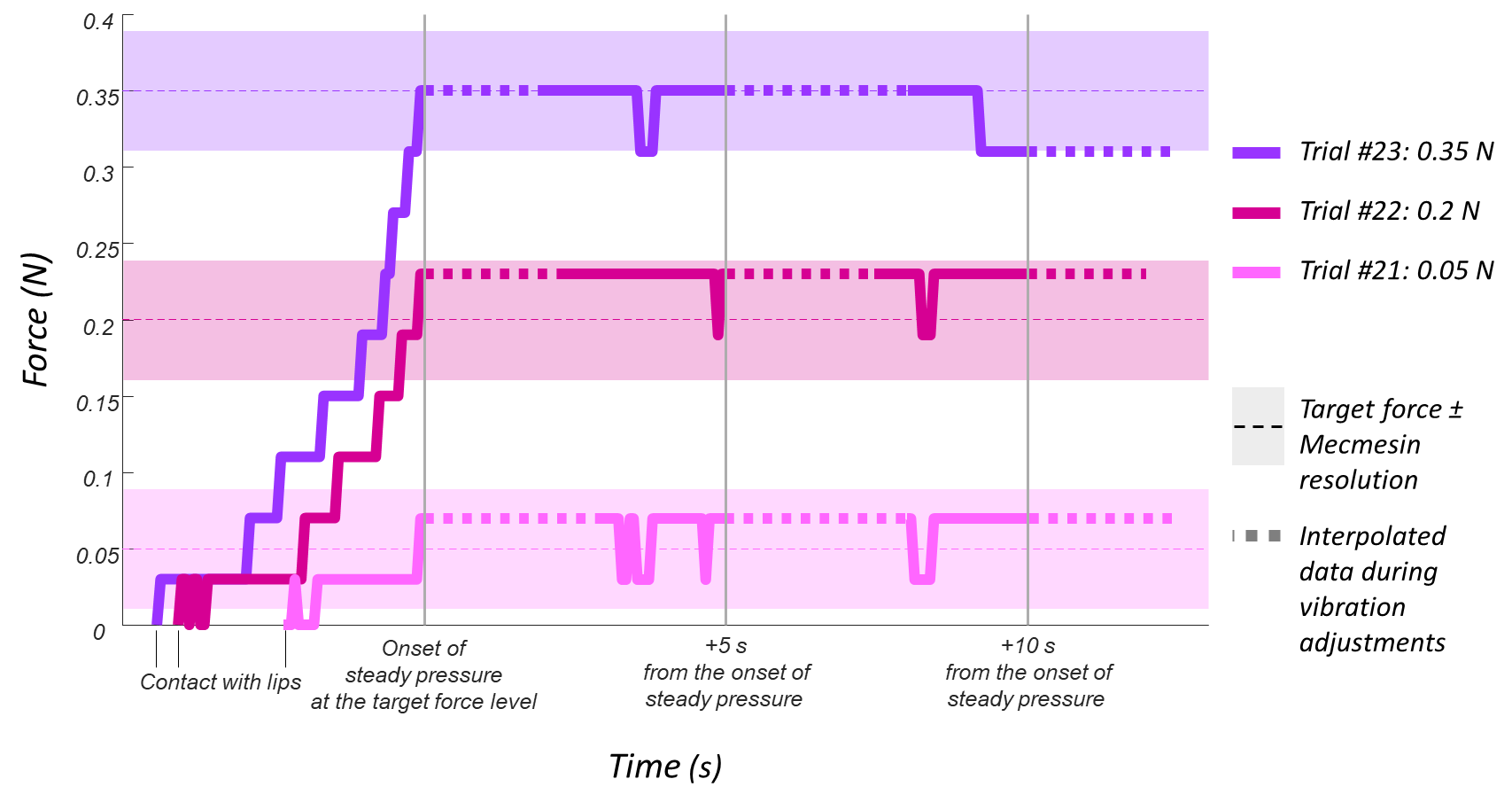


**Supplementary Figure S3. Force traces from three trials of a representative participant in Experiment 4.** The continuous coloured lines represent the reading of the Mecmesin force sensor from the moment of contact between the probe and the lips until the end of the trial. Three trials are shown, with force target levels of 0.35 N (purple trace), 0.2 N (red trace), and 0.05 N (pink trace). The dashed portions of each trace represent interpolated data for the period in which participants adjusted a mechanical vibration to the upper lip to match the intensity of sanshool-evoked tingle on the lower lip. During this adjustment period the force could not be recorded. The duration of each adjustment depended on the participant themselves (see Methods and Supplementary Video S1). Thin dashed horizontal lines indicate the target force for each trial. Surrounding coloured bands indicate the resolution of the Mecmesin PFI-200N (± 0.04 N) according to the manufacturer’s technical specification.


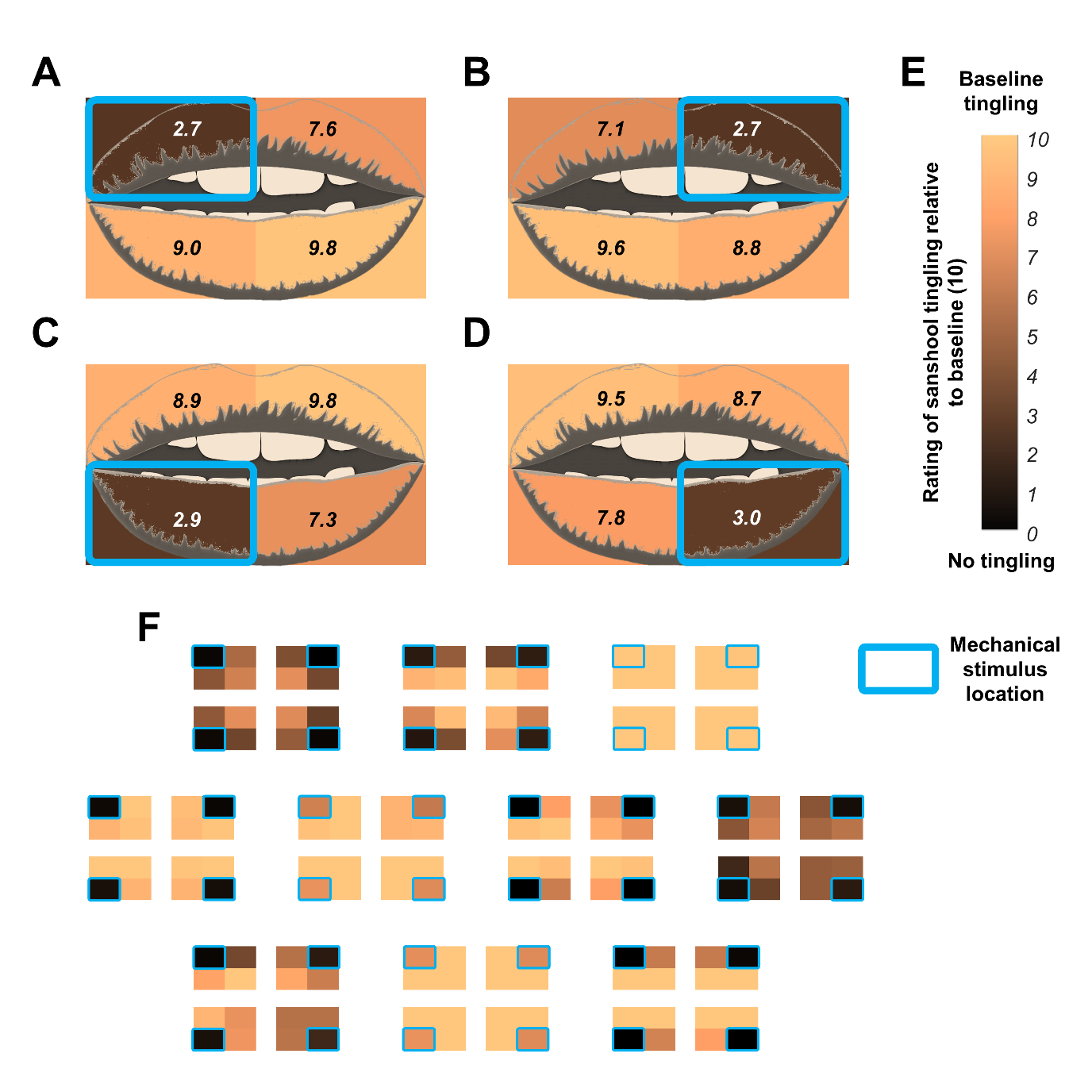


**Supplementary Figure S4: Reduction of sanshool tingling intensity on the lips in Experiment 2.** Sustained force was applied to different quadrants of the lip (**A**: Left upper, **B**: Right upper, **C**: Left lower, **D**: Right lower), while participants experienced sanshool induced tingling in all of the lip locations. Colour indicates the perceived intensity relative to the baseline period (**E**) (darker colours indicate lower ratings). **F**: Individual data. The tingling intensity was robustly inhibited at the quadrant where the sustained touch was applied, with less suppression in other, untouched quadrants.


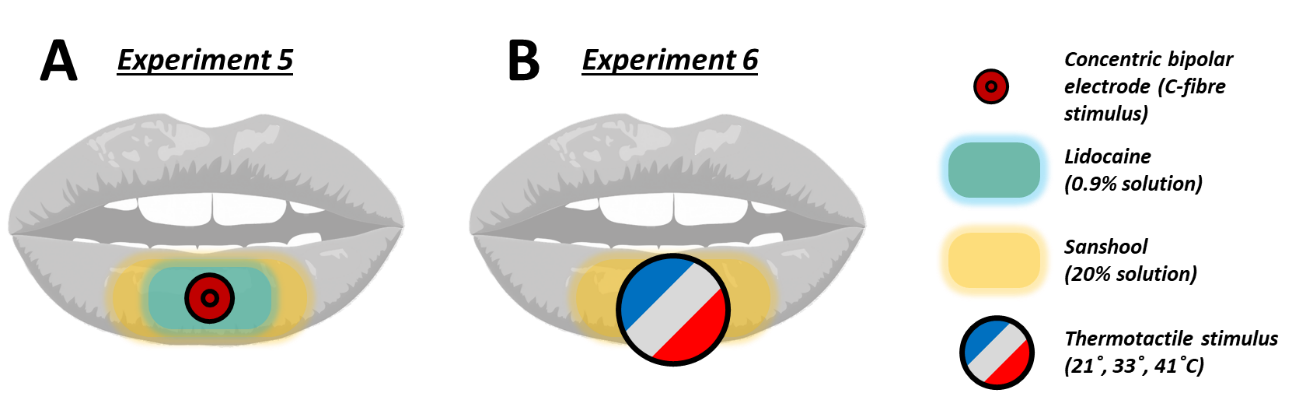


**Supplementary Figure S5. Experimental methods for Control Experiment 5 and 6.** **A:** In Control Experiment 5, the pain thresholds and tingling ratings of eight participants were measured during sanshool stimulation, both before and after topical application of Lidocaine (0.9%w/w). **B:** In Control Experiment 6, participants (n = 51) rated the intensity of sanshool tingling (20%) during three levels of thermotactile stimulation (21, 33, and 41° C).


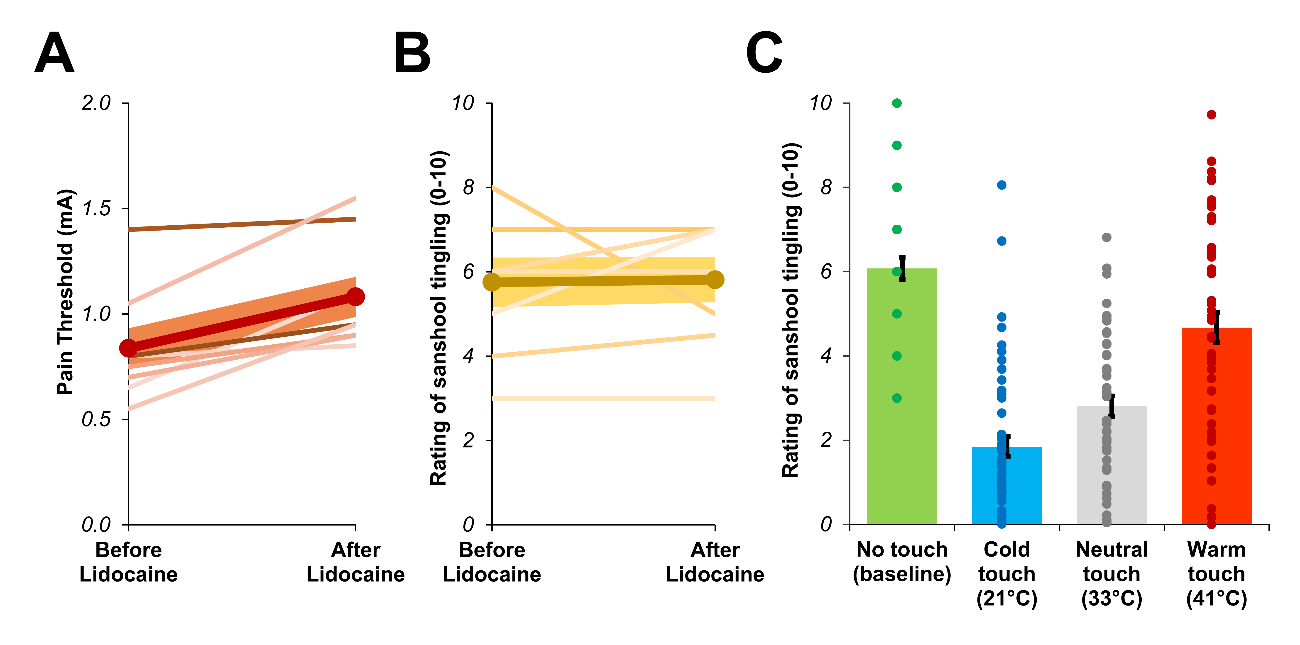


**Supplementary Figure S6. Results of Control Experiment 5 and 6.** **A-B:** Pain thresholds and ratings of sanshool tingling intensity before and after administration of lidocaine in Control Experiment 5 (n = 8). As expected, lidocaine induced a significant increase in participants’ pain thresholds (**A**). In contrast, the perceived intensity of sanshool-induced tingling was not affected by lidocaine administration (**B**). Dark lines represent the sample average, shadings represent the SEM, and coloured lines represent individual data. **C:** Participants’ ratings of sanshool-induced tingling during three thermo-tactile conditions in Control Experiment 6 (n = 51). The tingling intensity was linearly modulated by cold (21 °C), neutral (33 °C) and warm (41 °C) stimuli. Error bars indicate SEM across participants and coloured dots represent individual data.

**Supplementary Tables**

**Supplementary Table S1.** Table of means of Experiment 1.

|  | **Rating of tingling on the target position 6 when the position touched is:** | | | | | | | |
| --- | --- | --- | --- | --- | --- | --- | --- | --- |
| **Subj #** | **1** | **2** | **3** | **4** | **5** | **6** | **7** | **8** |
| **1** | 5.50 | 7.17 | 5.42 | 5.33 | 3.17 | 0.83 | 4.50 | 5.17 |
| **2** | 10.00 | 7.50 | 7.92 | 10.33 | 9.00 | 9.58 | 8.50 | 8.67 |
| **3** | 7.33 | 10.50 | 4.75 | 7.67 | 7.33 | 1.75 | 3.83 | 7.00 |
| **4** | 7.83 | 8.33 | 8.83 | 7.50 | 5.83 | 0.33 | 5.50 | 8.50 |
| **5** | 14.50 | 7.83 | 10.58 | 9.83 | 5.33 | 7.42 | 9.00 | 14.33 |
| **6** | 8.33 | 10.00 | 8.17 | 6.67 | 4.83 | 0.00 | 5.00 | 9.50 |
| **7** | 9.67 | 10.83 | 11.75 | 11.67 | 5.67 | 0.92 | 6.17 | 10.83 |
| **8** | 4.50 | 3.67 | 1.50 | 4.17 | 2.50 | 0.17 | 4.67 | 5.33 |
| **9** | 10.33 | 10.67 | 11.33 | 10.67 | 11.67 | 3.67 | 11.67 | 11.00 |
| **10** | 8.17 | 4.67 | 3.92 | 5.33 | 0.17 | 0.00 | 1.33 | 11.50 |

**Supplementary Table S2.** Table of means of Experiment 2.

|  | **Rating of tingling in each quadrant when position 1 is touched** | | | | **Rating of tingling in each quadrant when position 2 is touched** | | | | **Rating of tingling in each quadrant when position 3 is touched** | | | | **Rating of tingling in each quadrant when position 4 is touched** | | | |  |
| --- | --- | --- | --- | --- | --- | --- | --- | --- | --- | --- | --- | --- | --- | --- | --- | --- | --- |
| **S#** | **1** | **2** | **3** | **4** | **1** | **2** | **3** | **4** | **1** | **2** | **3** | **4** | **1** | **2** | **3** | **4** | |
| **1** | 0.3 | 5.3 | 4.3 | 6.5 | 4.0 | 0.0 | 7.2 | 3.7 | 4.5 | 7.2 | 0.5 | 3.5 | 7.0 | 3.2 | 4.7 | 0.3 | |
| **2** | 1.3 | 4.7 | 9.0 | 9.5 | 3.7 | 1.5 | 9.8 | 8.5 | 6.8 | 9.5 | 1.2 | 3.8 | 9.3 | 6.5 | 7.2 | 1.5 | |
| **3** | 10.0 | 10.3 | 10.3 | 11.3 | 10.5 | 9.8 | 11.2 | 11.0 | 10.7 | 10.0 | 10.0 | 11.7 | 10.7 | 11.7 | 10.8 | 10.3 | |
| **4** | 0.5 | 10.2 | 9.0 | 9.7 | 9.5 | 0.3 | 9.3 | 9.8 | 10.0 | 10.0 | 0.8 | 9.0 | 9.8 | 10.2 | 9.0 | 0.7 | |
| **5** | 6.5 | 10.0 | 9.7 | 10.0 | 9.0 | 6.2 | 9.0 | 9.2 | 10.0 | 10.0 | 7.3 | 10.0 | 10.0 | 10.0 | 10.0 | 7.0 | |
| **6** | 0.0 | 8.0 | 9.7 | 10.0 | 7.3 | 0.0 | 8.7 | 7.3 | 10.0 | 9.5 | 0.0 | 6.3 | 10.0 | 9.7 | 8.0 | 0.0 | |
| **7** | 0.8 | 6.2 | 4.3 | 6.7 | 4.3 | 0.7 | 5.2 | 5.8 | 2.0 | 5.8 | 0.7 | 3.3 | 4.7 | 4.8 | 4.7 | 1.3 | |
| **8** | 0.3 | 3.7 | 8.2 | 10.5 | 5.7 | 1.3 | 8.3 | 6.3 | 9.2 | 7.3 | 0.8 | 7.5 | 5.7 | 5.7 | 5.8 | 2.0 | |
| **9** | 7.2 | 11.0 | 10.3 | 10.3 | 10.3 | 7.0 | 10.7 | 10.7 | 11.8 | 12.2 | 7.3 | 11.0 | 12.0 | 11.3 | 10.3 | 7.0 | |
| **10** | 0.0 | 6.2 | 14.8 | 13.6 | 6.2 | 0.4 | 16.4 | 15.4 | 13.6 | 16.2 | 0.0 | 6.6 | 16.2 | 13.6 | 8.0 | 0.0 | |

**Supplementary Table S3.** Table of means of Experiment 3.

|  | **Probability (%) of reporting that the tingling on the lower lip is stronger than the tingling on the upper lip when force applied on the lower lip is:** | | | | |
| --- | --- | --- | --- | --- | --- |
| **Subj #** | **0.05 N** | **0.16 N** | **0.28 N** | **0.39 N** | **0.50 N** |
| **1** | 100.0 | 70.0 | 60.0 | 23.3 | 6.7 |
| **2** | 90.0 | 50.0 | 33.3 | 40.0 | 13.3 |
| **3** | 80.0 | 50.0 | 53.3 | 36.7 | 30.0 |
| **4** | 86.7 | 73.3 | 43.3 | 50.0 | 40.0 |
| **5** | 46.7 | 46.7 | 56.7 | 50.0 | 46.7 |
| **6** | 70.0 | 56.7 | 60.0 | 40.0 | 26.7 |
| **7** | 90.0 | 70.0 | 40.0 | 50.0 | 23.3 |
| **8** | 60.0 | 50.0 | 56.7 | 53.3 | 56.7 |

**Supplementary Table S4.** Table of means of Experiment 4. The data values correspond to the mechanical vibration amplitude perceived as equivalent to 80% Sanshool solution tingle (μm).

|  | **Baseline** | | | **Pressure onset**  **(0 s)** | | | **+5 s from pressure onset** | | | **+10 s from pressure onset** | | |
| --- | --- | --- | --- | --- | --- | --- | --- | --- | --- | --- | --- | --- |
| **Subj #** | **0.05 N** | **0.2**  **N** | **0.35**  **N** | **0.05 N** | **0.2**  **N** | **0.35**  **N** | **0.05 N** | **0.2**  **N** | **0.35**  **N** | **0.05 N** | **0.2**  **N** | **0.35**  **N** |
| **1** | 11.5 | 12.7 | 11.5 | 10.2 | 11.5 | 11.1 | 9.6 | 10.9 | 11.0 | 10.4 | 10.9 | 10.2 |
| **2** | 29.0 | 31.0 | 28.3 | 17.9 | 15.3 | 14.5 | 21.3 | 18.0 | 16.5 | 20.3 | 21.8 | 19.8 |
| **3** | 11.3 | 11.3 | 11.8 | 6.0 | 5.8 | 5.2 | 6.5 | 5.5 | 6.1 | 7.0 | 6.4 | 6.1 |
| **4** | 7.5 | 11.2 | 8.4 | 8.1 | 9.3 | 6.8 | 8.0 | 12.8 | 6.9 | 7.0 | 7.4 | 8.9 |
| **5** | 14.7 | 17.1 | 22.2 | 9.8 | 6.6 | 5.3 | 9.1 | 6.8 | 4.1 | 9.8 | 6.5 | 3.9 |
| **6** | 17.9 | 18.8 | 17.9 | 9.3 | 10.6 | 7.8 | 9.3 | 9.8 | 7.5 | 11.3 | 8.2 | 7.4 |
| **7** | 18.8 | 20.3 | 21.7 | 7.6 | 4.9 | 4.8 | 7.7 | 5.5 | 6.4 | 9.6 | 7.5 | 5.8 |
| **8** | 14.0 | 14.2 | 12.9 | 8.2 | 11.7 | 10.3 | 7.6 | 14.2 | 13.8 | 8.2 | 16.6 | 16.7 |
| **9** | 13.6 | 13.4 | 11.7 | 7.4 | 7.1 | 6.3 | 7.3 | 7.9 | 7.9 | 7.2 | 7.7 | 7.2 |
| **10** | 12.6 | 12.6 | 12.0 | 5.3 | 4.0 | 3.1 | 6.1 | 4.1 | 3.0 | 6.1 | 4.5 | 3.2 |
| **11** | 12.6 | 11.3 | 10.6 | 10.6 | 8.7 | 4.7 | 10.5 | 10.2 | 6.1 | 10.7 | 8.2 | 6.4 |
| **12** | 7.1 | 7.6 | 6.9 | 6.0 | 5.9 | 6.1 | 6.6 | 6.6 | 5.8 | 5.8 | 5.9 | 5.8 |
| **13** | 13.2 | 12.9 | 11.2 | 5.7 | 6.4 | 6.5 | 6.5 | 6.3 | 6.3 | 7.2 | 7.1 | 6.8 |
| **14** | 7.3 | 6.2 | 7.0 | 2.5 | 2.2 | 2.1 | 2.7 | 2.2 | 2.1 | 2.7 | 2.4 | 2.1 |

**Supplementary Table S5.** Table of individual data from Control Experiment 5.

| **Subj #** | **Pain threshold PRE (mA)** | **Pain threshold POST (mA)** | **Tingling rating PRE (0-10)** | **Tingling rating POST (0-10)** |
| --- | --- | --- | --- | --- |
| 1 | 0.8 | 0.9 | 7.0 | 7.0 |
| 2 | 0.7 | 0.9 | 7.0 | 7.0 |
| 3 | 1.1 | 1.6 | 8.0 | 5.0 |
| 4 | 0.6 | 1.0 | 4.0 | 4.5 |
| 5 | 0.8 | 0.9 | 6.0 | 7.0 |
| 6 | 0.7 | 1.1 | 6.0 | 6.0 |
| 7 | 0.8 | 1.0 | 3.0 | 3.0 |
| 8 | 1.4 | 1.5 | 5.0 | 7.0 |

**Supplementary Table S6.** Table of means of Control Experiment 6.

|  | | **Rating of tingling (VAS) when the tactile stimulus is at:** | | |
| --- | --- | --- | --- | --- |
| **Subj #** | **Baseline Tingle**  **(0-10)** | **21°C** | **33°C** | **41°C** |
| **1** | 4 | 0.0 | 0.1 | 5.2 |
| **2** | 3 | 6.7 | 3.5 | 4.9 |
| **3** | 5 | 2.0 | 3.6 | 6.1 |
| **4** | 3 | 3.0 | 4.3 | 7.7 |
| **5** | 4 | 0.3 | 1.8 | 2.0 |
| **6** | 7 | 1.0 | 1.8 | 4.5 |
| **7** | 8 | 3.1 | 6.1 | 7.2 |
| **8** | 6 | 1.2 | 0.7 | 2.2 |
| **9** | 5 | 3.2 | 5.0 | 8.6 |
| **10** | 6 | 2.6 | 4.8 | 7.5 |
| **11** | 5 | 0.6 | 0.9 | 0.0 |
| **12** | 10 | 1.1 | 4.6 | 5.3 |
| **13** | 4 | 1.2 | 2.0 | 2.2 |
| **14** | 3 | 1.5 | 6.8 | 9.7 |
| **15** | 10 | 0.0 | 0.2 | 0.2 |
| **16** | 6 | 4.9 | 5.9 | 7.6 |
| **17** | 6 | 1.5 | 2.0 | 7.3 |
| **18** | 8 | 0.9 | 3.1 | 3.9 |
| **19** | 4 | 4.7 | 2.4 | 6.5 |
| **20** | 6 | 0.2 | 1.3 | 1.3 |
| **21** | 3 | 0.7 | 1.3 | 3.5 |
| **22** | 7 | 0.1 | 1.7 | 2.4 |
| **23** | 8 | 1.2 | 4.3 | 2.0 |
| **24** | 5 | 1.6 | 2.2 | 4.9 |
| **25** | 7 | 1.8 | 0.5 | 1.3 |
| **26** | 6 | 4.1 | 4.9 | 6.0 |
| **27** | 5 | 0.9 | 4.3 | 6.4 |
| **28** | 8 | 1.2 | 3.0 | 8.2 |
| **29** | 10 | 0.1 | 2.0 | 6.4 |
| **30** | 8 | 3.9 | 2.0 | 3.9 |
| **31** | 4 | 0.7 | 4.8 | 8.2 |
| **32** | 8 | 0.2 | 3.1 | 4.4 |
| **33** | 4 | 3.4 | 5.2 | 7.3 |
| **34** | 8 | 1.5 | 0.9 | 0.4 |
| **35** | 7 | 8.1 | 4.0 | 3.7 |
| **36** | 6 | 2.1 | 3.2 | 4.9 |
| **37** | 7 | 0.8 | 0.9 | 4.1 |
| **38** | 6 | 4.3 | 2.4 | 5.1 |
| **39** | 9 | 1.4 | 3.7 | 8.4 |
| **40** | 8 | 3.7 | 3.1 | 2.7 |
| **41** | 6 | 0.8 | 2.2 | 1.6 |
| **42** | 4 | 0.6 | 0.1 | 4.0 |
| **43** | 7 | 1.5 | 4.9 | 3.2 |
| **44** | 8 | 1.7 | 2.0 | 2.7 |
| **45** | 6 | 0.3 | 0.0 | 0.2 |
| **46** | 6 | 1.8 | 4.0 | 6.1 |
| **47** | 6 | 1.1 | 4.5 | 6.5 |
| **48** | 5 | 0.1 | 1.8 | 7.7 |
| **49** | 6 | 2.1 | 1.5 | 1.0 |
| **50** | 6 | 1.9 | 2.5 | 6.6 |
| **51** | 3 | 0.7 | 0.6 | 4.9 |

**Supplementary Table S7. Normality test for Experiment 4.**

|  | **Shapiro-Wilk test** | | |
| --- | --- | --- | --- |
| **Condition** | **Statistic** | **df** | **p-value** |
| **0.05_Baseline** | 0.860 | 14 | 0.031 |
| **0.05_0s** | 0.887 | 14 | 0.073 |
| **0.05_5s** | 0.738 | 14 | 0.001 |
| **0.05_10s** | 0.843 | 14 | 0.018 |
| **0.2_Baseline** | 0.866 | 14 | 0.037 |
| **0.2_0s** | 0.970 | 14 | 0.877 |
| **0.2_5s** | 0.957 | 14 | 0.677 |
| **0.2_10s** | 0.799 | 14 | 0.005 |
| **0.35_Baseline** | 0.861 | 14 | 0.031 |
| **0.35_0s** | 0.919 | 14 | 0.214 |
| **0.35_5s** | 0.889 | 14 | 0.078 |
| **0.35_10s** | 0.833 | 14 | 0.013 |

**Supplementary Video S1**

A video showing the setup in Experiment 3 and 4, and an example trial from Experiment 4 can be found at <https://tinyurl.com/yyuoecqd>.

**Supplementary References**

1. Hagura N, Barber H, Haggard P. 2013 Food vibrations: Asian spice sets lips trembling. *Proceedings of the Royal Society B: Biological Sciences* **280**, 20131680–20131680. (doi:10.1098/rspb.2013.1680)

2. Kuroki S, Hagura N, Nishida S, Haggard P, Watanabe J. 2016 Sanshool on The Fingertip Interferes with Vibration Detection in a Rapidly-Adapting (RA) Tactile Channel. *PLOS ONE* **11**, e0165842. (doi:10.1371/journal.pone.0165842)

3. Bautista DM, Sigal YM, Milstein AD, Garrison JL, Zorn JA, Tsuruda PR, Nicoll RA, Julius D. 2008 Pungent agents from Szechuan peppers excite sensory neurons by inhibiting two-pore potassium channels. *Nat Neurosci* **11**, 772–779. (doi:10.1038/nn.2143)

4. Klein AH *et al.* 2011 A tingling sanshool derivative excites primary sensory neurons and elicits nocifensive behavior in rats. *J. Neurophysiol.* **105**, 1701–1710. (doi:10.1152/jn.00922.2010)

5. Lennertz RC, Tsunozaki M, Bautista DM, Stucky CL. 2010 Physiological basis of tingling paresthesia evoked by hydroxy-alpha-sanshool. *J. Neurosci.* **30**, 4353–4361. (doi:10.1523/JNEUROSCI.4666-09.2010)

6. Nagi SS *et al.* 2019 An ultrafast system for signaling mechanical pain in human skin. *Science Advances* **5**, eaaw1297. (doi:10.1126/sciadv.aaw1297)

7. Gokin AP, Philip B, Strichartz GR. 2001 Preferential Block of Small Myelinated Sensory and Motor Fibers by LidocaineIn VivoElectrophysiology in the Rat Sciatic Nerve. *Anesthesiology: The Journal of the American Society of Anesthesiologists* **95**, 1441–1454.

8. Sheets PL, Jarecki BW, Cummins TR. 2011 Lidocaine reduces the transition to slow inactivation in Nav1. 7 voltage-gated sodium channels. *British journal of pharmacology* **164**, 719–730.

9. Mouraux A, Iannetti GD, Plaghki L. 2010 Low intensity intra-epidermal electrical stimulation can activate Aδ-nociceptors selectively. *Pain* **150**, 199–207.

10. Paqueron X, Leguen M, Rosenthal D, Coriat P, Willer JC, Danziger N. 2003 The phenomenology of body image distortions induced by regional anaesthesia. *Brain* **126**, 702–712. (doi:10.1093/brain/awg063)

11. Albin KC, Simons CT. 2010 Psychophysical evaluation of a sanshool derivative (alkylamide) and the elucidation of mechanisms subserving tingle. *PLoS One* **5**, e9520.

12. Levitt H. 1971 Transformed up-down methods in psychoacoustics. *The Journal of the Acoustical society of America* **49**, 467–477.

13. McGlone F, Wessberg J, Olausson H akan. 2014 Discriminative and affective touch: sensing and feeling. *Neuron* **82**, 737–755.

14. Olausson H akan, Wessberg J, McGlone F, Vallbo \AAke. 2010 The neurophysiology of unmyelinated tactile afferents. *Neuroscience & Biobehavioral Reviews* **34**, 185–191.

15. Ackerley R, Wasling HB, Liljencrantz J, Olausson H akan, Johnson RD, Wessberg J. 2014 Human C-tactile afferents are tuned to the temperature of a skin-stroking caress. *Journal of Neuroscience* **34**, 2879–2883.

16. Löken LS, Wessberg J, McGlone F, Olausson H akan. 2009 Coding of pleasant touch by unmyelinated afferents in humans. *Nature neuroscience* **12**, 547.

17. Djouhri L. 2016 Electrophysiological evidence for the existence of a rare population of C-fiber low threshold mechanoreceptive (C-LTM) neurons in glabrous skin of the rat hindpaw. *Neuroscience Letters* **613**, 25–29. (doi:10.1016/j.neulet.2015.12.040)

18. Nagi SS, Mahns DA. 2013 Mechanical allodynia in human glabrous skin mediated by low-threshold cutaneous mechanoreceptors with unmyelinated fibres. *Exp Brain Res* **231**, 139–151. (doi:10.1007/s00221-013-3677-z)

19. Nagi SS, Dunn JS, Birznieks I, Vickery RM, Mahns DA. 2015 The effects of preferential A- and C-fibre blocks and T-type calcium channel antagonist on detection of low-force monofilaments in healthy human participants. *BMC Neuroscience* **16**, 52. (doi:10.1186/s12868-015-0190-2)

20. Rouder JN, Speckman PL, Sun D, Morey RD, Iverson G. 2009 Bayesian t tests for accepting and rejecting the null hypothesis. *Psychonomic bulletin & review* **16**, 225–237.

21. Wetzels R, Wagenmakers E-J. 2012 A default Bayesian hypothesis test for correlations and partial correlations. *Psychonomic Bulletin & Review* **19**, 1057–1064. (doi:10.3758/s13423-012-0295-x)
